# Mobilization of progenitor cells and vessel healing after implantation of SYNERGY in acute coronary syndrome

**DOI:** 10.1038/s41598-021-96730-9

**Published:** 2021-08-27

**Authors:** Masashi Sakuma, Setsu Nishino, Takahisa Nasuno, Michiya Kageyama, Michiaki Tokura, Ryoichi Sohma, Ken-ichi Inoue, Tomoaki Kanaya, Syotaro Obi, Shigeru Toyoda, Shichiro Abe, Guiherme F. Attizzani, Gabriel T. R. Pereira, Hiram G. Bezerra, Teruo Inoue

**Affiliations:** 1 Department of Cardiovascular Medicine, Dokkyo Medical University School of Medicine, 880 Kitakobayashi, Mibu, Tochigi 321-0293 Japan; 2 Advanced Medical Science Research Center, Dokkyo Medical University, Mibu, Tochigi Japan; 3 Division of Cardiovascular Medicine, Harrington Heart and Vascular Institute, University Hospitals Cleveland Medical Center, Case Western Reserve University School of Medicine, Cleveland, OH USA

**Keywords:** Cardiovascular biology, Interventional cardiology

## Abstract

This study was aimed to compare the vascular healing process of a SYNERGY stent with that of a PROMUS PREMIER stent in patients with acute coronary syndrome (ACS). In 71 patients with ACS, undergoing coronary stent implantation using the SYNERGY stent (n = 52) or PROMUS PREMIER stent (n = 19), we measured circulating CD34+/CD133+/CD45^null^ cells and CD34+/KDR+ cells and observed vascular healing at the stented sites using optical coherence tomography (OCT) and coronary angioscopy. On the day 7, circulating CD34+/CD133+/CD45^null^ cells increased in SYNERGY group (*P* < 0.0001), while it did not change in PROMUS group. The CD34+/KDR+ cells also increased in SYNERGY group (*P* < 0.0001) but less significantly in the PROMUS group (*P* < 0.05). The OCT-based neointimal thickness (*P* < 0.0005) and neointimal coverage rate (*P* < 0.05) at 12 months were greater in SYNERGY group, compared with PROMUS group. The coronary angioscopy-based neointimal coverage grade at 12 months was also greater in SYNERGY group (*P* < 0.001). In overall patients, the change in CD34+/KDR+ cells on the day 7 correlated with the OCT-based neointimal thickness at 12 months (R = 0.288, *P* < 0.05). SYNERGY stent seems to have potential advantages over PROMUS PREMIER stent for ACS patients in terms of vascular healing process at the stented sites.

## Introduction

Advances in drug-eluting stent (DES) technology have resulted in reduced target lesion revascularization across broad patient and lesion subsets. However, concerns over incomplete stent healing even with second generation DES persist^1^. Re-endothelialization and neointimal coverage over the stent struts are essential for vascular healing after stent deployment. In the process of vascular healing, endothelial (EPCs) as well as smooth muscle (SMPCs) progenitor cells are mobilized from bone marrow and other tissues into injured-vessel sites, possibly triggered by inflammatory response, and serve as a source of both smooth muscle cell and endothelial cell precursors in the healing response^2–4^.

**Figure 1:**
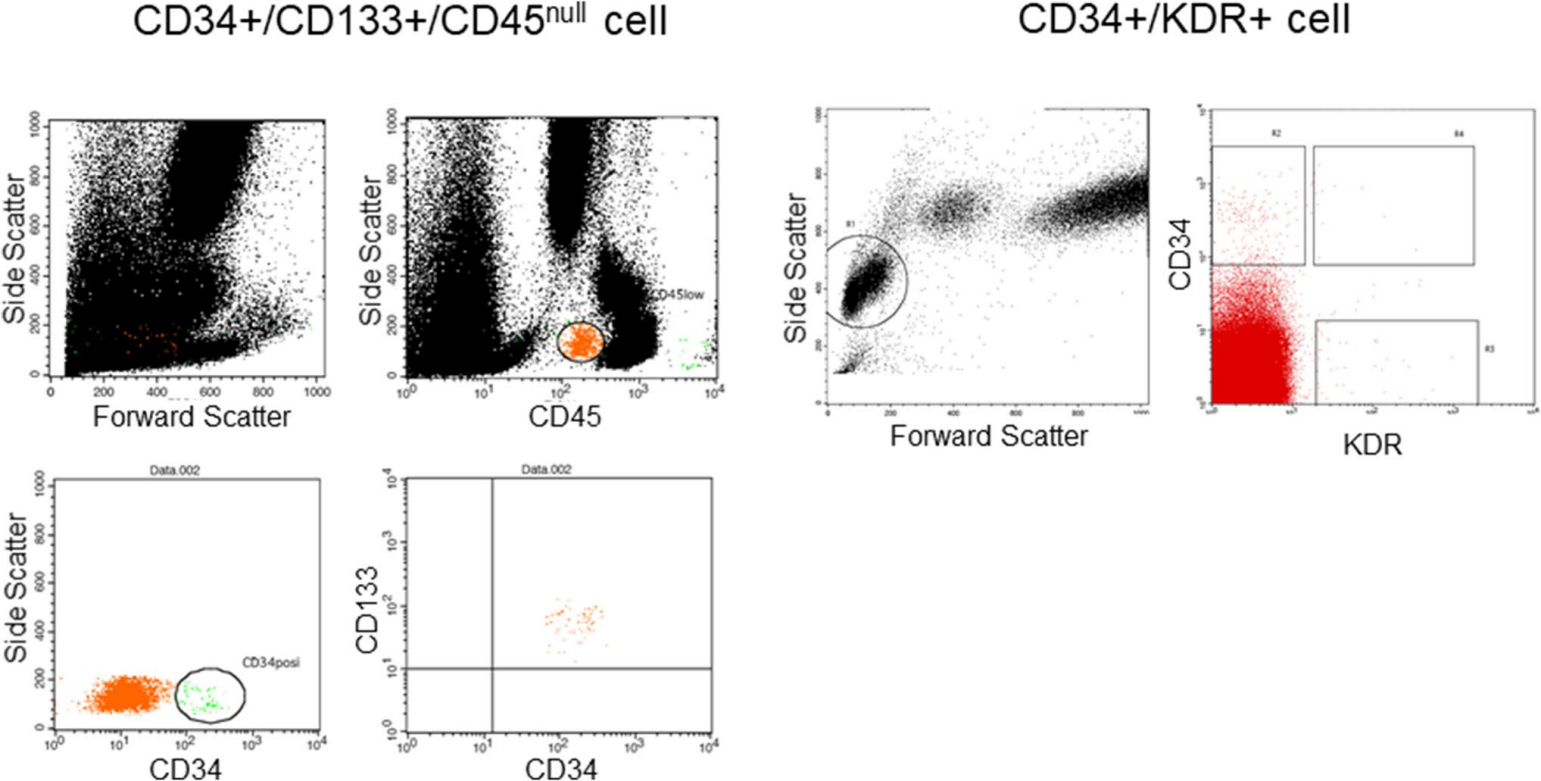
Detection of circulating CD34 +/CD133 +/CD45^null^ cells and CD34 +/KDR+ cells by flow cytomeric analysis. To detect the CD34+ CD133+ CD45^null^ cells, the mononuclear cell fraction was gated and analyzed for the expression of CD34 and CD45 cells. Only the CD34 +/CD45^null^ cells were finally investigated for the count of CD133+ cells. For detection of CD34 +/KDR+ cells, the CD34 and KDR double-positive fraction was detected after the gating of mononuclear cells.

A new generation DES stent, SYNERGY (Boston Scientific), consists of a thin strut (74 μm), balloon-expandable platinum-chromium stent platform delivering everolimus from an ultrathin (4 μm) bioabsorbable poly(d,l-lactide-co-glycolic acid) (PLGA) polymer applied to the abluminal surface, has been developed to target optimal vascular healing via its biological and pharmacological characteristics. In the present study, we observed mobilized progenitor cells and assessed the association between their kinetics and vascular healing at the stent-injured vessel sites in patients with acute coronary syndrome (ACS) who underwent emergent percutaneous coronary intervention (PCI) with coronary stent implantation, and compared the SYNERGY stent with the second generation durable polymer (polyvinylidene difluoride: PVDF) everolimus-eluting stent, PROMUS PREMIER (Boston Scientific) (Fig. 1).

## Results

### Baseline characteristics

Baseline characteristics were compared between two groups of SYNERGY and PROMUS (Table 1). Peak creatine kinase and triglyceride levels were higher in the SYNERGY group than in the PROMUS group. The other parameters were comparable between two groups. The stent implantation procedures were comparable between the two groups, except for the number of stent, which was more in the SYNERGY group than in the PROMUS group (Table 2).

**Table 1:** Baseline characteristics (full analyses set).

	SYNERGY group	PROMUS group	*p* value
	(n = 52)	(n = 19)	
Age, years	64 ± 10	66 ± 9	0.343
Male gender, n (%)	49 (94)	16 (84)	0.386
Body mass index, kg/m^2^	25 ± 3	24 ± 3	0.169
**Basal disease, n (%)**			
STEMI/ NSTEMI/ UA	33 (63)/ 14 (27)/ 5 (10)	8 (42)/ 9 (47)/ 28 (11)	0.241
**Target vessel, n (%)**			
LAD/ LCX/ RCA	33 (63)/ 6 (12)/ 13 (25)	8 (42)/ 2 (11)/ 9 (47)	0.208
**Affected vessel, n (%)**			0.792
Single vessel disease	38 (73)	13 (68)	
Multi-vessel disease	14 (27)	6 (32)	
Peak CK-MB, U/L	225 ± 244	113 ± 190	0.049
BNP, pg/mL	53 ± 60	93 ± 93	0.093
Ejection fraction, %	57 ± 6	60 ± 7	0.075
**Coronary risk factor**			
Hypertension, n (%)	33 (62)	16 (84)	0.052
Diabetes mellitus, n (%)	22 (42)	10 (53)	0.410
Dyslipidemia, n (%)	28 (54)	14 (74)	0.112
Current smoking, n (%)	26 (50)	11 (58)	0.514
LDL-cholesterol, mg/dL	125 ± 35	124 ± 37	0.853
HDL-cholesterol, mg/dL	46 ± 12	51 ± 10	0.085
Triglyceride, mg/dL	157 ± 94	98 ± 60	0.003
Hemoglobin A1c, %	6.2 ± 1.0	6.4 ± 1.1	0.604
eGFR, mL/min/1.73m^2^	78.4 ± 21.4	96.8 ± 43.3	0.094
hsCRP, mg/dL	0.16 ± 0.17	0.25 ± 0.38	0.311
**Medication, n (%)**			
Statins	52 (100)	19 (100)	1.000
ACE inhibitors/ARB	51 (98)	17 (89)	0.037
Anti-diabetic agents	19 (37)	7 (37)	0.740

STEMI: ST-elevation myocardial infarction; NSTEMI: non ST-elevation myocardial infarction; UA: unstable angina; LAD: left anterior descending artery; LCX: left circumflex artery, RCA: right coronary artery; CK-MB: creatine kinase-MB isoform; BNP: brain natriuretic peptide; LDL: low-density lipoprotein; HDL: high-density lipoprotein; eGFR: estimated glomerular filtration rate; hsCRP: high sensitivity C-reactive protein; ACE: angiotensin converting enzyme; ARB: angiotensin receptor blocker.

**Table 2:** Procedural characteristics.

	SYNERGY group (n = 52)	PROMUS group (n = 19)	*p* value
Number of stent, n	1.12 ± 0.33	1.00 ± 0.00	0.013
Total stent length, mm	26.7 ± 11	21.9 ± 8.2	0.054
Stent diameter, mm	3.46 ± 0.45	43.34 ± 0.47	0.360
Pre-dilatation, n (%)	27 (52)	7 (37)	0.295
Post-dilatation, n (%)	19 (37)	6 (32)	0.740
Maximum inflation pressure, atm	14 ± 3.8	14 ± 3.1	0.580
**Pre-procedural TIMI flow, n (%)**			0.834
Grade 0	29 (56)	9 (47)	
Grade 1	1 (2)	0 (0)	
Grade 2	7 (13)	4 (21)	
Grade 3	15 (29)	6 (32)	
**Post-procedural TIMI flow, n (%)**			0.773
Grade 0	0 (0)	0 (0)	
Grade 1	0 (0)	0 (0)	
Grade 2	2 (4)	0 (0)	
Grade 3	50 (96)	19 (100)	

TIMI: Thrombolysis in Myocardial Infarction.

### Circulating progenitor cells

**Figure 2:**
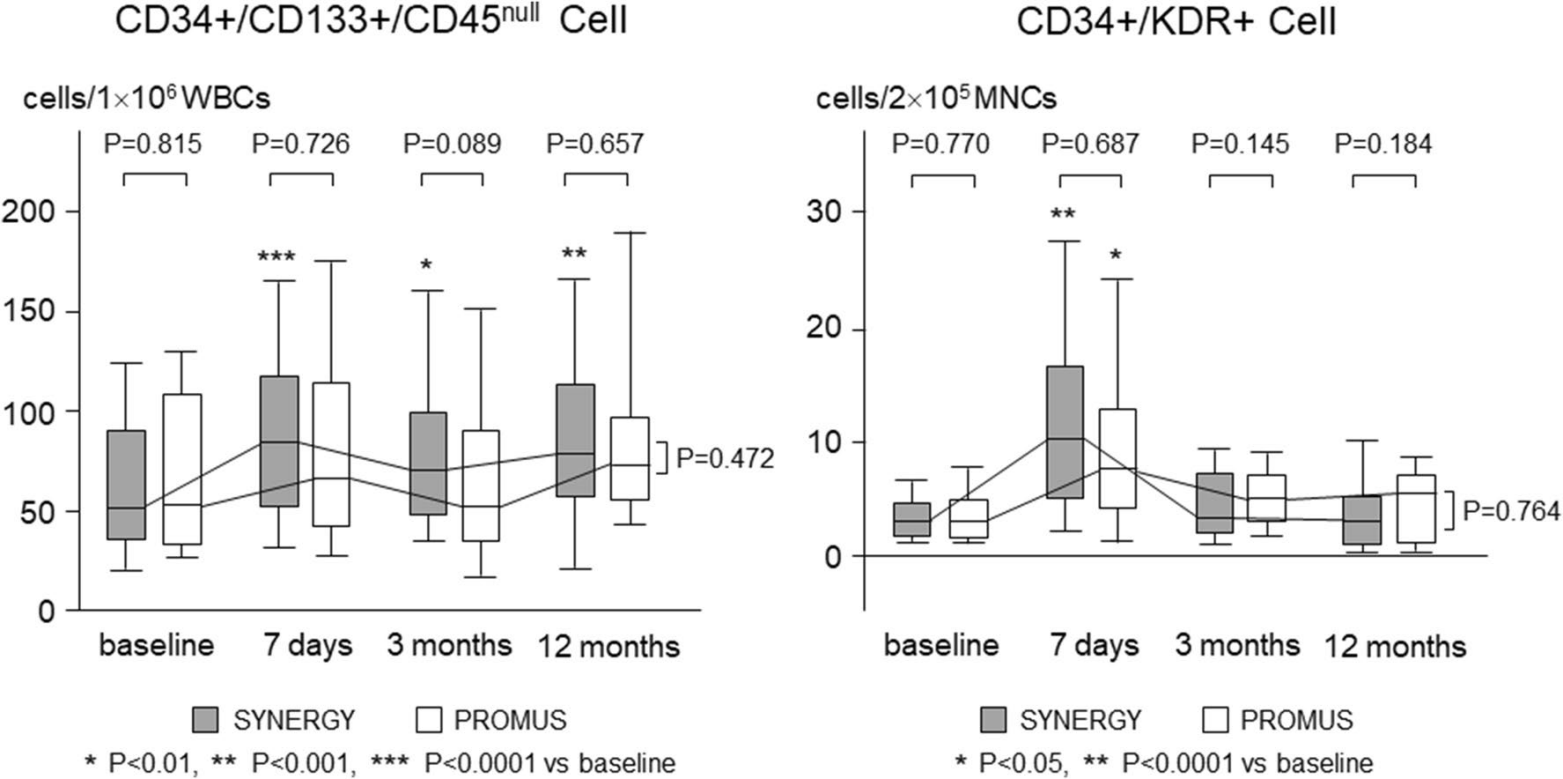
Comparison of serial changes in circulating progenitor cells during acute phase after stent implantation between 2 groups of SYNERGY and PROMUS.

**Figure 3:**
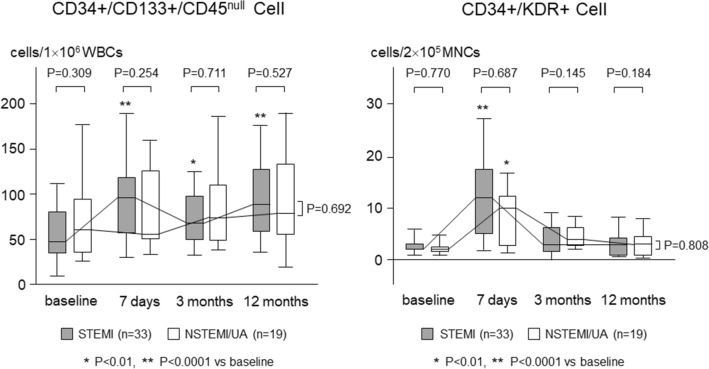
Comparison of serial changes in circulating progenitor cells between patients with ST-elevation myocardial infarction and those with non ST-elevation myocardial infarction or unstable angina in the limited patients of the SYNERGY group. STEMI: ST-elevation myocardial infarction; NSTEMI; non ST-elevation myocardial infarction; UA: unstable angina.

Serial changes in circulating CD34+/CD133+/CD45^null^ cells and CD34+/KDR+ cells are shown in Fig. 2. Baseline levels of CD34+/CD133+/CD45^null^ cells and CD34+/KDR+ cells were comparable between both groups of SYNERGY and PROMUS [CD34+/CD133+/CD45^null^ cells: 52 (35–91) and 51 (33–106) cell/1 × 10^6^ WBCs, respectively; CD34+/KDR+ cells: 3 (2–4) and 3 (2–5) cell/2 × 10^5^ MNCs, respectively]. The CD34+/CD133+/CD45^null^ cells significantly increased on the day 7 in the SYNERGY group [to 84 (52–117) cell/1 × 10^6^ WBCs, *P* < 0.0001], while it did not change in the PROMUS group [to 67 (43–114) cell/1 × 10^6^ WBCs]. The CD34+/KDR+ cells also significantly increased on the day 7 in the SYNERGY group [to 10 (5–16) cell/2 × 10^5^ MNCs, *P* < 0.0001], while it increased less significantly [to 8 (4–13) cell/2 × 10^5^ MNCs, *P* < 0.05] in the PROMUS group. In the limited 52 SYNERGY group patients, the CD34+/CD133+/CD45^null^ cells significantly increased on the day 7 in 33 patients with STEMI [48 (35–77) to 96 (58–117) cell/1 × 10^6^ WBCs, *P* < 0.0001], while it did not change in 19 patients with NSTEMI or UA [60 (36–93) to 55 (51–118) cell/1 × 10^6^ WBCs]. The CD34+/KDR+ cells also significantly increased on the day 7 in the STEMI patients [2 (3–3) to 12 (5–17) cell/2 × 10^5^ MNCs, *P* < 0.0001], while it increased less significantly [4 (2–5) to 7 (4–11) cell/2 × 10^5^ MNCs, *P* < 0.01] in the NSTEMI or UA patients (Fig. 3).

### Optical coherence tomographic and coronary angioscopic findings

In the OCT findings, percentage of uncovered struts to total struts and mean neointimal thickness were comparable between the two groups at 3 months follow-up coronary angiography. At 12 months follow-up, however, the percentage of uncovered struts was less (*P* < 0.05) and the mean neointimal thickness was greater (*P* < 0.001) in the SYNERGY group, compared with the PROMUS group (Table 3).

**Table 3:** Optical coherence tomography findings.

	SYNERGY group	PROMUS group	
	(n = 52)	(n = 19)	*p* value
**3 months follow-up**			
Covered struts, %	82.8 ± 12.3	85.9 ± 12.3	0.350
Uncovered struts, %	17.2 ± 12.3	14.1 ± 12.3	0.351
Malaposed Struts, %	0.65 ± 1.13	0.33 ± 0.68	0.147
Mean neointimal thickness, mm	0.07 ± 0.03	0.07 ± 0.02	0.521
**12 months follow-up**			
Covered struts, %	98.0 ± 3.8	92.5 ± 9.8	0.027
Uncovered struts, %	1.97 ± 3.75	7.50 ± 9.80	0.027
Malaposed Struts, %	0.73 ± 1.84	0.44 ± 0.73	0.337
Mean neointimal thickness, mm	0.16 ± 0.06	0.11 ± 0.04	0.0001

**Figure 4:**
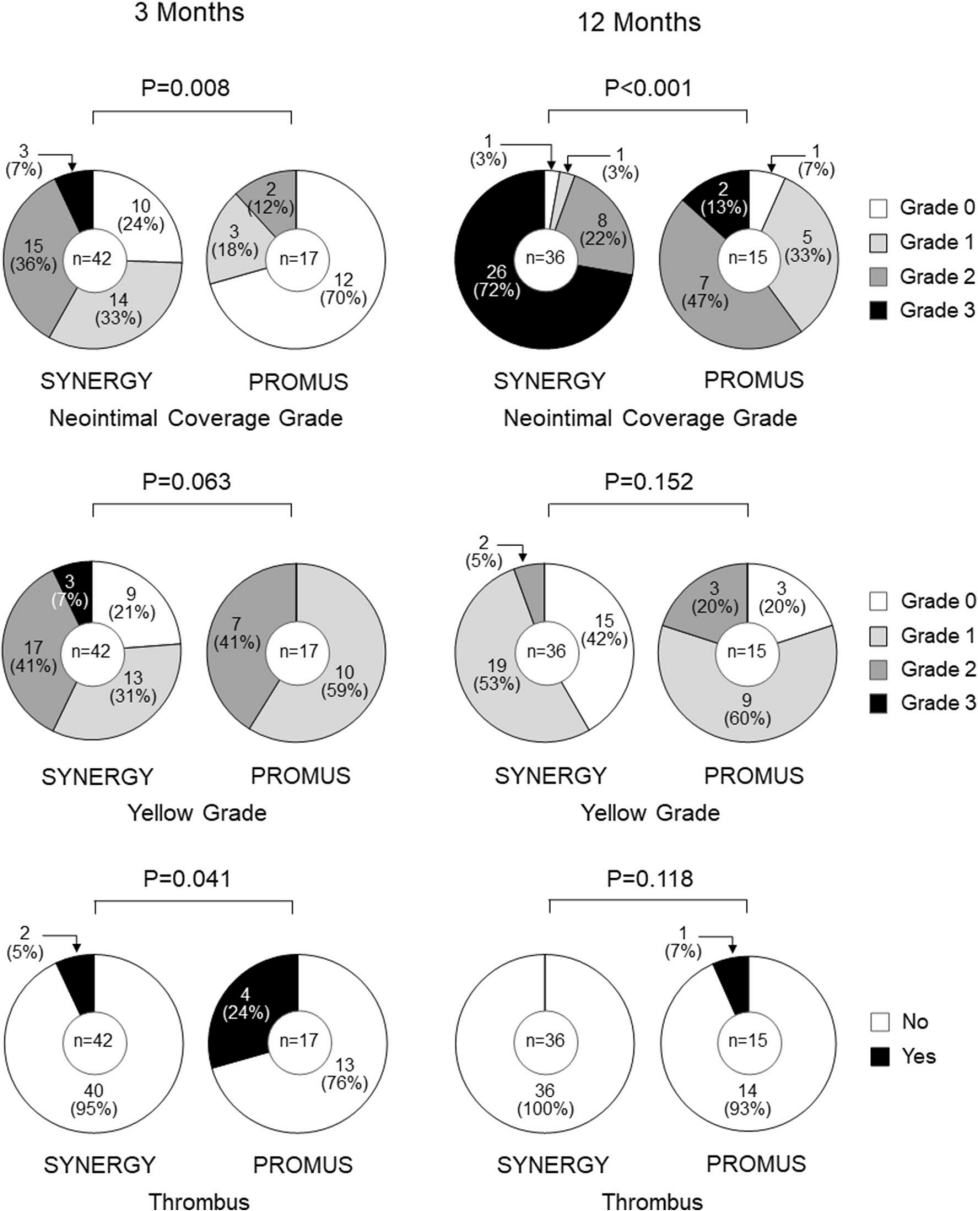
Neointimal coverage grade, yellow color grade and thrombus formation at 3 and 12 months by coronary angioscopy.

In the coronary angioscopic findings, neointimal coverage grade was higher (*P* < 0.05) and thrombus was less present (*P* < 0.05) in the SYNERGY group than in the PROMUS group at the 3 months follow-up. At the 12 months follow-up, neointimal coverage grade was still higher in the SYNERGY group than in the PROMUS group, although presence of thrombus was comparable between the two groups. Yellow color grade was comparable between the two groups at both 3 months and 12 months (Fig. 4).

**Figure 5:**
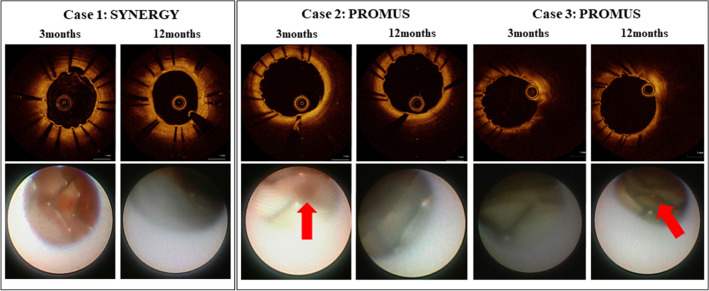
Representative OCT and coronary angioscopic findings. Red arrows: thrombus.

Representative OCT and coronary angioscopic findings are shown in Fig. 5. In a case of the SYNERGY group (Case 1), although uncovered or malapposed struts were observed at 3 months follow-up, the struts were completely covered by white neointima (neointimal coverage grade 3) at 12 months follow-up. On the other hand, in a case of the PROMUS group (Case 2), mural thrombus in addition to the uncovered stent struts were observed at 3 months follow-up, and neointimal coverage grade was still 0–1 at 12 months follow-up. In another case of the PROMUS group (Case 3), mural thrombus, uncovered struts (neointimal coverage grade 1) and yellow neointima (yellow grade 2) was observed even at 12 months follow-up.

### Association between mobilization of progenitor cells and neointima formation

**Figure 6:**
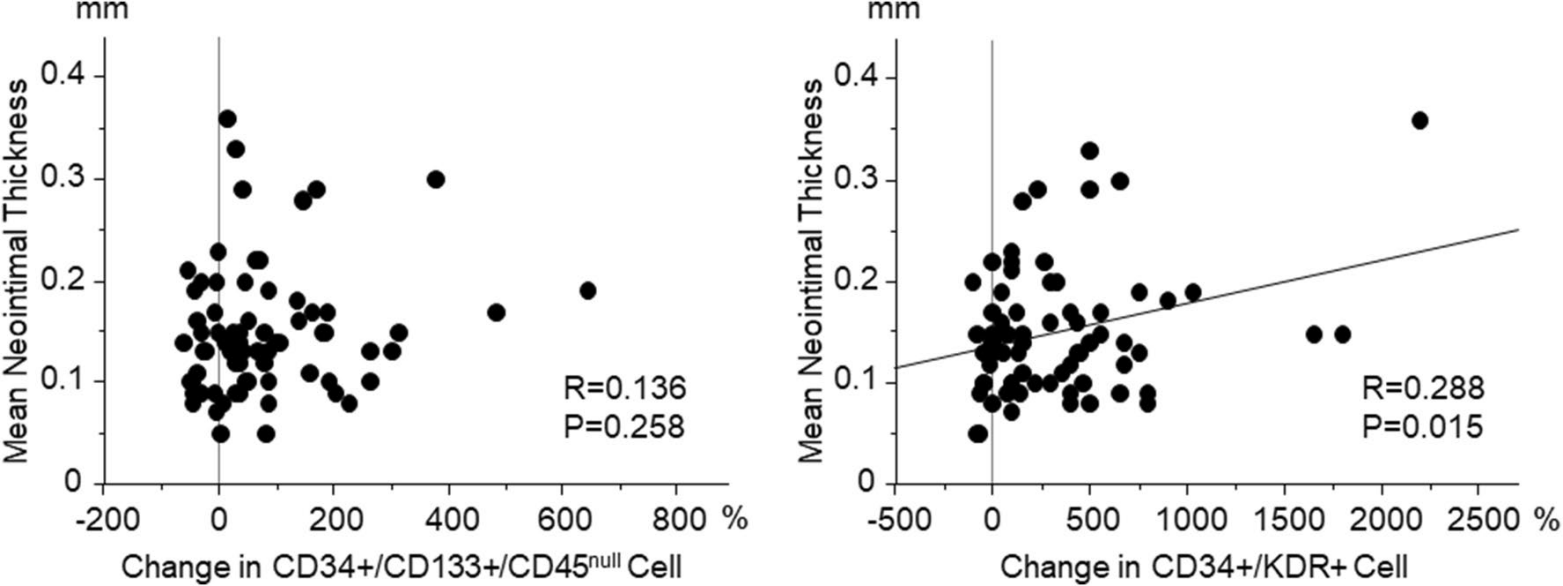
Correlation between kinetics of progenitor cells and OCT-based stent healing in all of 71 patients combined SYNERGY and PROMUS groups.

In all 71 patients combined in the SYNERGY and PROMUS groups, there was no relationship between percent change in circulating CD34+/CD133+/CD45^null^ cells on the day 7 from the baseline values and OCT-based mean neointimal thickness at 12 months follow-up coronary angiography (R = 0.136). However, the mean neointimal thickness at 12 months follow-up was correlated with percent change in CD34+/KDR+ cells on the day 7 (R = 0.288, *P* < 0.05) (Fig. 6).

### Clinical outcomes

**Figure 7:**
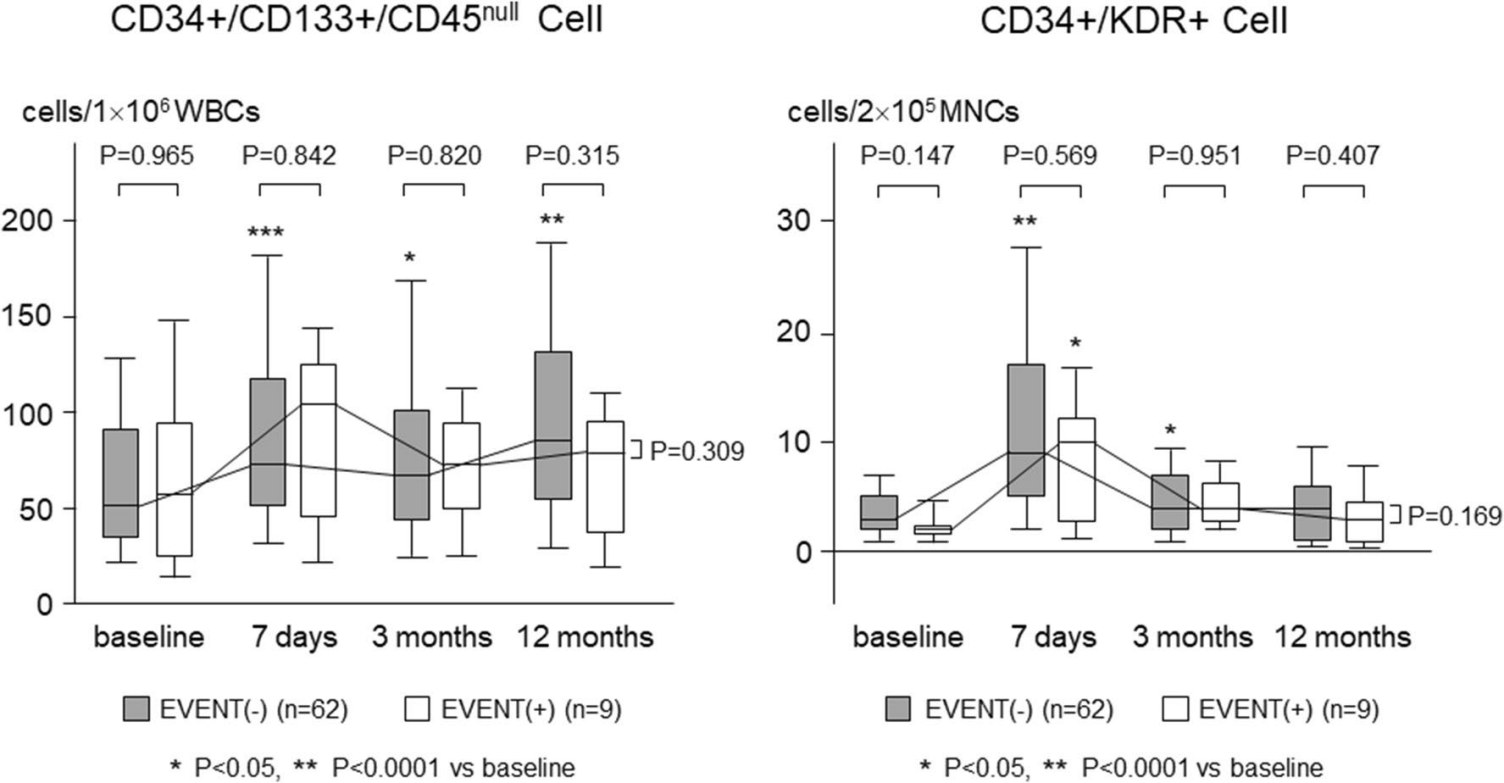
Comparison of serial changes in circulating progenitor cells between patients who experienced the event such as revascularization other than target lesion at 12 months follow-up and those who did not.

There were no cardiovascular events in both groups of PROMUS and SYNERGY at 3 months. At 12 months, revascularization other than target lesion was shown in 2 patients (11%) of the PROMUS group and in 7 patients (13%) of the SYNERGY group (*P* = 0.742). The other cardiovascular events were absent in both groups. In all of 71 both group patients, serial changes in the progenitor cells were assessed separately in 9 patients who experienced the event, i.e., revascularization other than target lesion, at 12 months follow-up and in 62 patients who did not. As a result, CD34+/CD133+/CD45^null^ cells significantly increased on the day 7 in the patients without event [51 (34–91) to 70 (51–117) cell/1 × 10^6^ WBCs, *P* < 0.0001], while it did not change in the patients with event [57 (26–91) to 104 (51–122) cell/1 × 10^6^ WBCs]. The CD34+/KDR+ cells also significantly increased on the day 7 in the patients without event [3 (2–5) to 9 (5–17) cell/2 × 10^5^ MNCs, *P* < 0.0001], while it increased less significantly [2 (1–3) to 10 (3–12) cell/2 × 10^5^ MNCs, *P* < 0.05] in the patients with event (Fig. 7).

## Discussion

In the present study, we demonstrated in patients with ACS that circulating CD34+/CD133+/CD45^null^ cells and CD34+/KDR+ cells increased significantly on the day 7 in the SYNERGY group, while the CD34+/CD133+/CD45^null^ cells did not change and the CD34+/KDR+ cells increased less significantly in the PROMUS group. In addition, our OCT findings showed significantly less percentage of uncovered struts and significantly greater mean neointimal thickness at 12 months in the SYNERGY group, compared with PROMUS group. The coronary angioscopy findings showed that neointimal coverage grade was higher and thrombus was less present in the SYNERGY group than in the PROMUS group at the 3 months follow-up. At 12 months follow-up, neointimal coverage grade was still higher in the SYNERGY group than in the PROMUS group. Interestingly, the mean neointimal thickness at 12 months was correlated with percent change in CD34+/KDR+ cells on the day 7, in combined patients of both SYNERGY and PROMUS groups. These results suggest that the SYNERGY stent might have some advantage over the PROMUS PREMIER stent, in terms of stent-induced mobilization of progenitor cells and subsequent healing of stent-injured vessel sites, in patients with ACS.

### Novel concept stent, SYNERGY

Future DES technology requires a novel concept, optimizing vascular healing^5^. In the process of vascular healing at stent-injured vessel sites, re-endothelialization and subsequent neointima formation are essential^6^. The neointimal stent coverage and maturation of endothelial cells depend on metal alloy, stent strut thickness, polymer composition, and polymer bioresorption^7^. In this regard, the SYNERGY stent was designed to promote and to enhance stent healing. Several clinical trials demonstrated the safety and efficacy of SYNERGY stent in a broad range of patients undergoing percutaneous coronary intervention^8–10^. On the other hand, more favorable vascular healing of SYNERGY stent has been directly observed by advanced imaging modalities such as OCT ^11,12^ or coronary angioscopy^13^, compared with the second generation DESs. In the present study, vascular healing measured by neointima formation, stent coverage and anti-thrombogenicity was compared between the newer generation DES, SYNERGY stent and the second generation DES, PROMUS PREMIER stent, uniquely using both imaging modalities of OCT and coronary angioscopy at 3 and 12 months. The results of both the OCT and coronary angioscopy findings might indicate potential vascular healing advantages of the SYNERGY stent over the second generation DES stents, supporting previous data. In particular, a noteworthy finding is the coronary angioscopy result that demonstrates better vascular healing was evident in the SYNERGY stent even at the 3 months follow-up.

### Vascular injury and endothelial progenitor cells

The biological response to stent-induced vascular injury is characterized by a cascade of cellular events, including endothelial denudation, platelet deposition, leukocyte recruitment and accumulation, smooth muscle cell proliferation and migration, and the deposition of extracellular matrix proteins^2^. After coronary stent implantation, EPCs mobilize from bone marrow and other tissues, possibly triggered by inflammatory response, migrate to sites of stent-induced vascular injury and differentiate into endothelial cells, contributing in part to re-endothelialization and ultimately stent strut coverage, i.e., vascular healing^2–4^. Previously, we observed that bone marrow-derived progenitor cells including EPCs were mobilized maximally on the day 7 after stent implantation, leading to vascular healing in patients with stable coronary artery disease (CAD) undergoing implantation of bare metal stents. In our observation, however, DESs suppressed mobilization of the progenitor cells and -limus analogues, such as everolimus as well as sirolimus, suppressed differentiation of EPCs into vascular endothelial cells^14,15^.

In the present study, we used flow cytometric marker CD34+/KDR+ cells, in addition to CD34+/CD133+/CD45^null^ cells, both of which abundantly include EPC lineage^16,17^. As a result, a stronger mobilization of both CD34+/CD133+/CD45^null^ cells and CD34+/KDR+ cells was induced at early stage in the SYNERGY group, compared with the PROMUS group, possibly associated with more favorable vessel healing at late stage after the SNYERGY stent implantation. The result of correlation between percent change in CD34+/KDR+ cells at early stage and OCT-based neointimal thickness at late stage indicates that CD34+/KDR+ cells might be associated with wound healing response at the stent-injured vessel sites. We believe that our findings of progenitor cell kinetics strongly support advantages of SYNERGY stent for vascular healing.

### Stent healing in acute coronary syndrome

The biological response to stent-induced vascular injury and subsequent healing mechanism in patients with ACS may be different from those in stable CAD patients. Therefore, use of DES stents for ACS should be discussed, separately from that for stable CAD. Because of concerns around inadequate vascular healing, initially the use of DES had not been recommended in the context of ACS, which possesses higher risk of stent thrombosis than stable CAD, in the first generation DES era^18,19^. However, such a risk has been reduced in the second generation DESs^20^. In regard to the new generation SYNERGY stent versus second generation DESs, there are no event-driven clinical trials, but the OCT findings demonstrated more favorable vascular healing after SYNERGY stent implantation, compared with the second generation DES also in patients with ACS^12^.

In our previous observations for mobilization of progenitor cells in the vascular healing process after stent implantation, subjects were stable CAD patients^14,15^. In the present study, we selected ACS patients for the subjects to assess kinetics of progenitor cells after coronary stent implantation for unstable or ruptured plaques, in which local inflammatory reaction is accelerated even at baseline before the procedures. Consequently the CD34+/CD133+/CD45^null^ cells significantly increased on the day 7 even for the second generation PROMUS PREMIER stent, which we selected as a control, contrary to our previous results for the second generation DES in stable CAD patients^15^. The difference in kinetics of these cells between ACS and stable CAD might be based on the difference in baseline plaque-related inflammatory status at the stented sites, because mobilization of the progenitor cells might be triggered by inflammatory reaction. In addition, ACS included acute myocardial infarction, i.e., STEMI and NSTEMI, in which infarct itself might evoke strong inflammation. Therefore, mobilization of the progenitor cells might depend not only on plaque-related and/or stent-induced vascular inflammatory reaction but also homing induced by infarct itself. In our results, increase in circulating CD34+/CD133+/CD45^null^ cells and CD34+/KDR+ cells on the day 7 was more striking in the STEMI patients, compared with the NSTEMI or UA patients, in the limited patients of the SYNERGY group. The result suggests that infarct itself induced in part the homing of the progenitor cells, because infarct size is generally larger in STEMI, compared with NSTEMI. In the present study, the percentage of STEMI patients was higher in the SYNERGY group, compared with PROMUS group, although the difference was not statistically significant (63% vs 42%) (Table 1). And thus, the possibility that the difference of STEMI proportion affected the different kinetics of circulating CD34+/KDR+ cells between both groups cannot be denied. However, our result of correlation between CD34+/KDR+ cell kinetics and OCT-based neointimal thickness might strongly suggest a role of progenitor cells on wound healing response at the stent-injured vessel sites. Taken together, from our results we can envision that a new generation SYNERGY stent may produce advantageous vascular healing over the second generation DES also in patients with ACS. Finally, in the present study, clinical outcomes at 3 months and 12 months follow-up were also assessed and resulted that an event, revascularization other than target lesion, was shown in 9 patients (7 patients in the SYNERGY group and 2 in the PROMUS group). Interestingly, increases in circulating CD34+/CD133 +/CD45^null^ cells and CD34 +/KDR+ cells on the day 7 were more striking in the patients without event, compared with those with event. Although the result suggests that the kinetics of circulating progenitor cells potentially predict clinical outcomes, the number of patients as well as events was too small and the observation period was too short to conclude.

### Study limitation

The major limitation of our study is the study design. Although we compared mobilization of progenitor cells and subsequent vascular healing at stented sites between SYNERGY stent and PROMUS PREMIER stent, the comparison was not performed with the randomized design. We designed this study in the beginning of 2015, when we were using the PROMUS PREMIER stent for ACS patients and had an information that the SYNERGY stent would become available since in February 2016. Consequently, the subject recruitment was performed from April 2015 to January 2016 for the PROMUS arm, and thereafter started at February 2016 for the SYNERGY arm. Therefore, the number of patients were imbalanced between each group of PROMUS PREMIER (n = 19) and SYNERGY (n = 52) and the gender seemed imbalance, although 94 vs 84% of male was not statistically significant (*P* = 0.386). In order to match the number of subjects in both groups, we considered the option of ‘Matched Analysis,’ but we avoided it because sample size would be extremely small if the number of subjects was matched in both groups. In the present study, we assessed clinical outcomes only at 3 months and 12 months and could only an event such as revascularization other than target lesion, because the other cardiovascular events were absent in the limited number of patients and in the limited observation period. To establish the clinical importance of treatment, a larger scale event-driven trial with longer term observation period would be required. In the present study, we selected patients with ACS, including STEMI, NSTEMI and UA. To identify whether the mobilization of progenitor cells is due to the plaque-related and/or stent-induced vascular inflammation or to the homing induced by infarct itself, we should set the study design as a comparison with untreated patients or stable CAD patients as the controls. Although the comparison with untreated patients is not realistic because almost ACS patients undergo PCI in current clinical practice, that with stable CAD patients would be helpful. Finally, in the present study, we investigated only the quantitative changes in circulating progenitor cells using flow cytometric analysis. In the present study, we assessed progenitor cells using the CD34 +/CD133 +/CD45^null^ cells and CD34 +/KDR+ cells. de Bore, et al.^21^ has demonstrated that the circulating CD34 +/KDR+ cells are not mobilized from bone marrow as a predestined endothelial progenitor cell population but are mostly generated from circulating multipotent CD34+ cells at sites of vascular injury and act as tissue repair cells. Therefore, the CD34 +/KDR+ cells might represent more functional cells, compared with other CD34+ cell fractions. Since there have been no reports that assessed relationship between CD34 +/KDR+ cell mobilization and OCT-based or angioscopy-based vessel healing in ACS, we believe that our study has a certain novelty in that regard. Still yet, however, to assess the role of progenitor cells on vascular healing process, it would be important to examine the function of these cells and their angiogenic potential more profoundly.

Although the present study has several potential weaknesses, we believe our data would have some value in terms of representing real-world clinical practice.

### Clinical implication/conclusion

Impaired vascular healing at stented sites is associated with a risk of stent thrombosis, especially in DESs implantation. Despite generational advances in DES technology altering the healing responses, the controversy around dual anti-platelet therapy (DAPT) duration after implantation of DES remains in patients with ACS as well as stable CAD. Even for ACS patients, attempts for shorter DAPT duration is being explored in the new generation DES era, but such data are insufficient^20,22^. From our data, we can envision that shorter duration DAPT would be promising with the usage of SYNERGY stent.

Recent EPC-capturing stent technology is an ultimate technology to promote and to enhance vascular healing, in terms of our concept demonstrated by the present study^23^. Future advances in next generation stents targeting physiological vascular healing would be promising.

We observed in this study that mobilization of progenitor cells was more remarkable and subsequent neointima formation and stent coverage were better in the SYNERGY stent than in the PROMUS PREMIER stent. The results suggest that the SYNERGY stent seems to have potential advantages over the PROMUS PREMIER stent for ACS patients in terms of vascular healing process at the stented sites.

## Methods

### Study design

This study was designed to be conducted within the scope of daily medical practice. In the early 2015, we used only PROMUS PREMIER as the second generation DES. Around that time, we had an information that the third generation DES, SYNERGY, would be available in Japan at the beginning of 2016. At that time, we planned this study. We made the plan to correct data regarding PROMUS PREMIER until SYNERGY became available, and after that, to collect SYNERGY data. Consequently, the study included 71 consecutive patients with ACS, who underwent emergent PCI with coronary stent implantation for an ACS-related lesion during the periods between April 2015 and the end of 2017. From April 2015 to January 2016, the PROMUS PREMIER stent was implanted in 19 patients (PROMUS group; 16 men and 3 women, aged 66 ± 9 yr). The SYNERGY stent was implanted in 52 patients (SYNERGY group; 49 men and 3 women, aged 64 ± 10 yr) from February 2016, when the SYNERGY stent became available in our hospital, to the end of 2017. The ACS was defined as follows: presence of chest discomfort or ischemic symptoms lasting ≥ 10 min, ST-segment deviation ≥ 1 mm, or T-wave inversion ≥ 3 mm, or elevated levels of biomarkers for myocardial necrosis, including ST-elevation myocardial infarction (STEMI), non ST-elevation myocardial infarction (NSTEMI) and unstable angina (UA). Patients with complications of culprit lesion of left main trunk, acute heart failure of Killip III or IV, serious arrhythmia, and/or systemic complications including infectious diseases, chronic kidney disease over stage III and diabetes mellitus requiring insulin injection, were excluded.

The follow-up coronary angiography was performed for all patients at 3 months and 12 months after stent implantation. All of the patients received dual anti-platelet therapy with 81 mg of aspirin and 75 mg clopidogrel until the follow-up coronary angiography at 12 months. In all patients, peripheral blood sample was collected at baseline before stent implantation, on the day 7 post-implantation, and at the time of 3 and 12 months follow-up coronary angiography. The blood samples were immediately collected into tubes containing ethylene diaminetetraacetate (EDTA). We serially measured the number of circulating progenitor cells using the EDTA blood. At 3 and 12 months follow-up coronary angiography, we assessed re-endothelialization and neointima formation at the site of stent placement using two imaging modalities such as optical coherence tomography (OCT) and coronary angioscopy.

The local institutional review board in Dokkyo Medical University (Mibu, Tochigi, Japan) approved the study protocol, and written informed consent was obtained from each patient.

### Measurement of progenitor cells

We measured circulating CD34 +/CD133 +/CD45^null^ and CD34 +/kinase insert domain receptor (KDR)+ progenitor cells as EPC linage, using flow cytometry based on a previously described method^16,17^ with minor modifications. In brief, EDTA-treated peripheral blood (3 ml) was incubated with test reagent or control reagent. The reagent mixture consisted of a nucleic acid dye (SY-III-8; Molecular Probe), a peridinine chlorophil protein (PerCP)-conjugated anti-CD45 (Becton Dickinson), a fluorescein isothiocyanate (FITC)-conjugated anti-CD34 (Becton Dickinson), a phycoerythrin (PE)-conjugated anti-CD133 (Miltennyi Biotec) and PE-conjugated anti-KDR (Becton Dickinson). Isotype controls were used as negative controls based on the species and immunoglobulin (Ig) G control antibodies (IgG1 isotype control; Becton Dickinson). The samples were incubated for 20 min at room temperature and after incubation diluted with FACS-lysing solution (Becton Dickinson) for hemolysis. Flow cytometric analysis was then performed using the FACS Calibur laser flow cytometer (Becton Dickinson) according to the manufacturerʼs instructions. Measurement for CD34 +/CD133 +/CD45^null^ and CD34 +/KDR+ cells consisted of 1 × 10^6^ events of all white blood cells (WBCs) and 2 × 10^5^ events of mononuclear cells (MNCs), respectively, which exceeded a threshold set on SY-III-8 fluorescence (nucleated cells). The absolute cell number was calculated for both CD34 +/CD133 +/CD45^null^ cells and CD34 +/KDR+ cells (Fig. 1). To minimize any methodological variations, each sample was analyzed with two independent experiments, and the mean value was calculated.

### Optical coherence tomography imaging and analysis

At the time of follow-up coronary angiography, OCT examination was performed using a frequency-domain system (C7-XR FD-OCT Intravascular Imaging System; St Jude Medical). Cross-sectional OCT images were analyzed at 0.6 mm intervals. In every cross-sectional image, neointimal coverage was assessed for all of the struts, including uncovered struts and malapposed struts. Malapposed struts were defined by the distance between the endoluminal surface of the neointima and the strut ≤ 0 µm. The percentage of uncovered struts to total struts in all OCT cross-sections was then calculated. The cross-sections with major side branches (diameter ≥ 2 mm) were excluded from analysis^24^. Strut-level quantitative analysis was performed using all analyzable frames (0.6-mm intervals) along the stented segment. Strut-level intimal thickness was determined based on automated measurements performed from the center of the luminal surface of each strut blooming and its distance to the lumen contour^25^. Struts covered by tissue had positive intimal thickness values, whereas uncovered or malapposed struts had negative intimal thickness values. Mean neointimal thickness was calculated as an average of the intima thickness for all measured struts. The OCT analysis was performed by an independent investigator blinded to the study protocol (Cardiovascular Imaging Core Laboratory, Harrington Heart & Vascular Institute, University Hospitals, Cleveland Medical Center, OH, USA).

### Coronary angioscopy and semi-quantitative analysis

Coronary angioscopy was performed using a non-occlusive angioscope system (Visible; Intertec Medicals). The optical fiber was placed at the distal segment of the coronary artery and then carefully pulled back from the distal edge of the stent to the proximal edge. Branch vessels and luminal shapes were utilized as landmarks in order to ensure the same location corresponding with OCT images. The angioscopic images were obtained under an injection of low molecular weight dextran for cleaning red blood cells from imaging site. We assessed grade of neointimal coverage over the stent struts, color of the in-stent segment assessed as the yellow grade, and mural thrombi. Neointimal coverage was classified into 4 grades as previously described: grade 0, stent struts exposed; grade 1, struts bulging into the lumen although covered; grade 2, struts embedded but seen translucently; and grade 3, struts fully embedded and invisible on angioscopy. The yellow color grade was also classified into 4 grades visibly based on the surface color as previously reported: grade 0, white; grade 1, light yellow; grade 2, medium yellow; and grade 3, dark yellow^26,27^. The exact position of the angioscopic catheter at the stent site was recorded by an angiogram. All angioscopic images were analyzed by observers who were blinded to the study design.

### Clinical outcomes

Clinical outcomes were also assessed at 3 months and 12 months follow-up coronary angiography, regarding cardiovascular events such as cardiovascular death, non-fatal myocardial infarction, non-fatal stroke, unstable angina requiring hospitalization, target lesion revascularization, and revascularization other than target lesion.

### Statistical analyses

Normality of the distribution of variables was assessed using Kolmogorov–Smirnov test with Lilliefors’ correlation. Values were presented as the means ± standard deviation for parametric data, and median values and interquartile ranges for non-parametric data. Intergroup comparisons were performed using unpaired t tests for parametric data and Mann Whitney’s U tests for non-parametric data. Intragroup comparisons were assessed using paired t tests for parametric data and Wilcoxon Rank Sum tests for non-parametric data. Serial changes in parameters were analyzed using repeated measures analysis of variance. Spearman correlation analyses were used to assess the relationship between 2 parameters. Categorical variables were compared using chi-square tests. *P* < 0.05 was considered to be significant.

### Human subjects/informed consent statement

All procedures followed were in accordance with the ethical standards of the responsible committee on human experimentation (institutional and national) and with the Helsinki Declaration of 1975, as revised in 2000 (5). Informed consent was obtained from all patients for being included in the study.
